# How does social support influence tourist-oriented citizenship behavior? A self-determination theory perspective

**DOI:** 10.3389/fpsyg.2022.1043520

**Published:** 2022-10-25

**Authors:** Ruyou Li, Zhangyu Shi

**Affiliations:** ^1^School of Humanities and Social Sciences, Yancheng Institute of Technology, Yancheng, Jiangsu, China; ^2^Department of Marketing and Tourism Management, Zhejiang Gongshang University Hangzhou College of Commerce, Hangzhou, China

**Keywords:** tourist-oriented citizenship behavior, self-determination theory, stimulus-organism-response model, information support, emotional support

## Abstract

As a driver of tourist-oriented citizenship behavior (TOCB), the effect of social support has not been thoroughly investigated. Grounded in a framework integrating the stimulus-organism-response model and self-determination theory, this study investigates how social support influences TOCB through the sense of self-determination. Structural equation modeling is used to analyze the survey data collected from 377 tourists in China. It is found that social support (information and emotional support) have a remarkably positive impact on the sense of self-determination (perceived autonomy, competence, and relatedness) which have an intermediary role in the relationship between social support and TOCB. This study provides empirical evidence for the marketing strategies of tourism destinations and enterprises.

## Introduction

Customer citizenship behavior has made an enormous contribution to the service quality, performance, and competitiveness of enterprises (Groth, 2005). Given the importance of customer citizenship behavior, scholars have applied this concept to the tourist sector (e.g., Liu and Tsaur, 2014; Cheng et al., 2016; Assiouras et al., 2019; Shafiee et al., 2020; Liu et al., 2021; Torres-Moraga et al., 2021; Tsaur et al., 2021). Building on Groth’s (2005) definition of customer citizenship behavior, tourist citizenship behavior is defined as tourists’ autonomously altruistic behaviors (Liu and Tsaur, 2014; Arica and Çorbaci, 2020). Many scholars have explored the antecedents of tourist citizenship behaviors (e.g., Cheng et al., 2016; Assiouras et al., 2019; Shafiee et al., 2020). Many studies have adopted the old understanding of customer citizenship behavior but have overlooked the differences in customer citizenship behavior in different industries, which precluded their findings from fully reflecting the characteristics of tourism activity (Liu et al., 2021).

Furthermore, tourist citizenship behavior consists of tourist-and destination-oriented citizenship behavior (Torres-Moraga et al., 2021). The two dimensions of tourist citizenship behavior are driven by different factors, as some scholars have recognized. However, most existing research focused on the factors influencing tourist citizenship behavior at the destination level or in the tour group (Torres-Moraga et al., 2021; Li et al., 2022; Zhang et al., 2022). In this context, our understanding of how tourist citizenship behavior is formed is incomplete, and much attention should be paid to the antecedent variables affecting TOCB.

Research has shown that people are embedded in a social network composed of relationships (Granovetter, 1983). These social relationships contain rich information and group norms that can affect every member’s cognition, preferences, and behaviors. Tourists’ decisions rely not only on their own information sets; their attitudes will be corrected by others’ words and deeds. Especially in ambiguous situations, tourists usually make decisions by relying on the views of closely related groups (Kang and Schuett, 2013). For example, when tourists face uncertainty (e.g., about service reliability), they will actively seek credible information sources, looking at the behavior of other tourists for clues as to how to behave (Yi et al., 2013). Thus, tourism activities far exceed the scope of temporary stay or consumption by tourists at their destinations. Tourism activity is the reproduction of individual social relations. Tourist consumption behaviors will inevitably be affected by social support from others (Kim and Tussyadiah, 2013; Chung et al., 2017).

Studies have found that customer behavior maintains a close association with social networks, and kindness from related individuals can stimulate customer citizenship behavior (Verleye et al., 2014; Chiu et al., 2015), or positively influence customer citizenship behavior by increasing satisfaction (Rosenbaum and Massiah, 2007; Zhu et al., 2016). Since customer citizenship behaviors usually refer to the source of benefits for individuals, social support, as the external benefits obtained by individuals in social networks, have a noticeable impact on individual customer citizenship behavior toward other customers (Yi et al., 2013). Tourist citizenship behavior is often induced from favorable encounters, as this reciprocity maintains a mutually beneficial relationship between tourists and operators (Chen et al., 2015). The quality of social interaction between tourists also influences tourist citizenship behavioral intention (Wong and Lin, 2022). However, few scholars have discussed how social support affects tourist citizenship behavior. To fill this gap, this study attempts to uncover the effect mechanism of social support on TOCB.

Self-determination theory is a cognitive motivation theory constructed on people’s needs for self-determination. According to this theory, humans are viewed as striving toward growth and optimal development, not just shaped by social learning or stimulus–response pairing. However, in order to attain optimal results, people require some positive support from the environment. The theory provides a new perspective for understanding the formation mechanism of TOCB and better explaining why and how factors identified by existing research influence TOCB. Based on self-determination theory, this study draws on the stimulus-organism-response (S-O-R) theory to interpret the effect of social support on TOCB and the intermediary role of self-determination to deepen our understanding of this behavior.

In brief, this study aims to explore how social support affects TOCB through the sense of self-determination. Specifically, the three specific objectives are as follows: (a) to explore the influences of social support on the sense of self-determination; (b) to investigate the effects of the sense of self-determination on TOCB; and (c) to examine the mediating role of the sense of self-determination in the social support–TOCB relationship. The comprehensive framework developed in this study contributes to the body of knowledge by extending the driving factors of TOCB from the perspective of individual psychology. In addition, the “black box” of the social support–TOCB relationship is described by comprehensively considering the sense of self-determination as a mediator. In practice, this study provides references for formulating marketing strategies for tourist destinations and enterprises.

## Theoretical background

### Social support

In social psychology, social support is the verbal and non-verbal communication between a provider and a recipient. Social support could reduce the uncertainty of situations, egos, others, or relationships and enhance the individual’s sense of control in the experience (Albrecht and Adelman, 1987). According to this definition, social support needs a provider, a recipient, and the transfer of resources. Social support is a prevalent social behavior (Thoits, 1995). Family members, friends, and colleagues are essential providers of social support, and anyone can receive it (Van der Poel, 1993). Strong social support can enable individuals to feel connected with other members of social networks and can build trust between people (Crocker and Canevello, 2008). A consumer market is an important place for customers to obtain social support, where enterprises, service staff, or even other consumers can provide social support (Hajli, 2014). In tourism consumption, social support may come from strong ties with friends or family (Klooster and Go, 2006) or weak ties with service providers, fellow travelers, and others with the same tourism experiences (Huang and Hsu, 2010). The social network is also an important channel for tourists to get social support (Kim and Tussyadiah, 2013).

Social support is a dimensional concept whose structure varies with the situation (Madjar, 2008; Chiu et al., 2015). Early researchers have identified three kinds of social support: information, emotional, and tangible (Schaefer et al., 1981). With the rapid development of information and communication technology, the boundary of social support has expanded from traditional support networks based on kinship and geographical relationships to flexible social support with the internet as media. In the era of the mobile internet, people can socialize face-to-face or on a virtual network. Information and emotional support become the main objectives of support on social networks. As a result, many scholars focus on intangible social support, which is based on the social network, and divide social support into information and emotional support (Liang et al., 2011; Hajli, 2014; Zhu et al., 2016). Information support focuses on information provided for recipients through advice, opinions, and knowledge to help solve problems. Emotional support concentrates on news delivered to recipients to express care, encouragement, and empathy. In tourism consumption, social support is manifested as providing tourists with information that can assist in arranging tourism activities and expressing emotions that can help strengthen their tourism experience. Thus, this study will analyze social support in terms of information and emotional support.

### Tourist-oriented citizenship behavior

The concept of tourist citizenship behavior was adopted from the customer citizenship behavior literature. Customer citizenship behavior is the spontaneous behavior of customers. It is not necessary but beneficial to the whole service organization in the process of transactions or service delivery (Groth, 2005). The tourism experience involves close contact between tourists and others before and after traveling (Frías Jamilena et al., 2017). In contrast, general customer citizenship behavior ignores the dynamic characteristics of social change in this continuous contact so that it cannot fully explain citizenship behavior (Liu and Tsaur, 2014). Therefore, it is necessary to conduct an in-depth discussion based on the unique situation of tourism consumption.

Liu and Tsaur (2014) expand the concept of customer citizenship behavior to tourist citizenship behavior and define it as autonomous altruistic behavior of the visitors in tourism activities, such as enlivening a team atmosphere, sharing personal resources, and doing service providers a favor. These behaviors benefit communication and management in the tourism team and can bring harmony to the team members (Tsaur et al., 2021).

The particularity of the tourism consumption situation determines the uniqueness of tourist citizenship behavior (Wong and Lin, 2022). Customer citizenship behavior focuses on consumers who support the business, while tourist citizenship behavior focuses on tourists who help the destination and other tourists (Torres-Moraga et al., 2021). In addition, tourist citizenship behavior is a vital part of tourism activities. If tourists get a sense of connection with the destination, they will define themselves by this feeling (Hultman et al., 2015; Kumar and Nayak, 2019), resulting in citizenship behaviors such as sharing, recommending, and helping (Rather et al., 2020). Tourist citizenship behavior regulates and guides the behavior of other tourists. While traveling, some tourists inadvertently violate social norms because they are not familiar with the culture of the tourist destination or have bad consumption habits. Tourist citizenship behavior can guide behavior through event presentations and codes of conduct (Liu and Tsaur, 2014).

As a discretionary behavior that is reciprocal in nature, tourist citizenship behavior is driven by tourist internal psychological and external environmental factors. Factors such as resource uniqueness, service quality, destination identification, perceived value, tour guide humor, and the quality of tourist-tourist interaction are considered the antecedents of tourist citizenship behavior (Liu et al., 2021; Torres-Moraga et al., 2021; Li et al., 2022; Wong and Lin, 2022; Zhang et al., 2022).

Tourist citizenship behavior is also dimensional. The two kinds of tourist citizenship behavior are TOCB and destination-oriented citizenship behavior (Torres-Moraga et al., 2021). The former direct beneficiaries are either actual or potential tourists, and the latter natural beneficiaries are the enterprises and residents in tourist destinations. Some scholars see three dimensions of tourist citizenship behavior: help, recommendation, and feedback (Li et al., 2022). In fact, help and recommendation should belong to TOCB because they are more directed to other tourists. In contrast, since feedback behavior refers to service providers, it should be the content of destination-oriented citizenship behavior.

Internet technology has changed the information collection methods of tourists, and the information shared by individual social circles is considered reliable (Kang and Schuett, 2013). Tourist sharing behavior has become an important part of TOCB, significantly affecting other tourists’ decisions and consumption behavior. Consequently, the dimensions of TOCB should be sharing, help, and recommendation. Sharing indicates the consumption experiences and experiences about tourism shared by tourists in their social circles. Help refers to providing assistance to optimize decisions or solve problems for others in need. Recommendation means that tourists advise others to visit a tourist destination.

### Self-determination theory and sense of self-determination

Proposed by Deci and Ryan in the 1980s, self-determination theory illustrates that individual behavior is voluntary and self-determining, based on the assumption that human beings tend to pursue psychological growth and development (Deci and Ryan, 2000). Deci and Ryan have proposed a theoretical system of five branches (Ryan and Deci, 2017). According to psychological needs theory, individuals’ three basic psychological needs are autonomy, competence, and relatedness. Autonomy means that an individual, when performing an activity, desires to make his or her own choices. Competence means that an individual wants to feel effective when interacting with the environment. Relatedness means that an individual wants to establish connections with others to obtain understanding, support, and respect from others. These needs are inborn and prevalent. However, they differ in the extent to which they are met (Gagné and Deci, 2005). Environmental factors can activate or awaken them. At the same time, individuals strive to meet these needs and tend to the environment that can meet these needs.

According to self-determination theory, every behavior in an individual can be explained in terms of whether a need has been satisfied. Individuals develop perceived autonomy, competence, and relatedness based on the degree to which these needs have been met psychological needs. Perceived autonomy denotes how much the individual’s behavior depends on their own will. Perceived competence is concerned with whether the individual is qualified for a task. Perceived relatedness posits that an individual is integrated into an environment and can feel cared for, valued, and relied on (Hsieh and Chang, 2016). If a social climate supports and satisfies the three needs, individuals will obtain a strong sense of self-determination and choose to participate in some activities beneficial to their own development and that of others (Engström and Elg, 2015). Self-determination theory defines the three basic psychological needs and illustrates the effect of environmental factors on inner motivation, which is an excellent theoretical vision for positive behavior. Many scholars have explored tourist self-expression behavior (Bosnjak et al., 2016), tourism participation behavior (Aicher et al., 2015), and tourist citizenship behavior (Liu et al., 2021) based on this theory, which shows the effectiveness and applicability of the tourism research field theory. Therefore, this study will investigate the effect mechanism of social support on TOCB based on self-determination theory.

## Conceptual model and hypotheses

### Research framework

**Figure 1:**
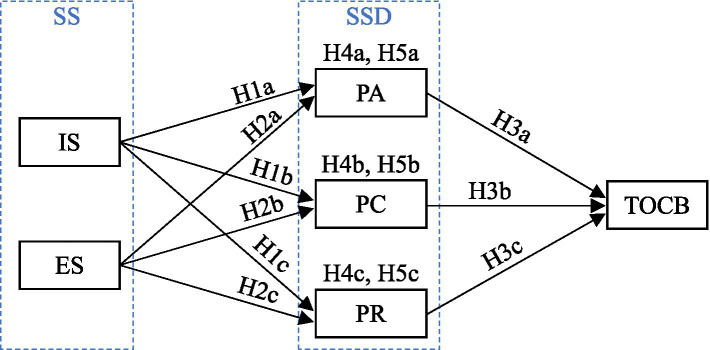
Conceptual framework. (1) SS, social support; SSD, the sense of self-determination; IS, information support; ES, emotional support; PA, perceived autonomy; PC, perceived competence; PR, perceived relatedness; TOCB, tourist-oriented citizenship behavior. (2) H4a: IS → PA → TOCB; H4b: IS → PC → TOCB; H4c: IS → PA → TOCB; H5a: ES → PA → TOCB; H5b: ES → PC → TOCB; H5c: ES → PA → TOCB.

The S-O-R analytical framework emphasizes that environmental factors will affect an individual’s mental status and elicit a behavioral response (Namkung and Jang, 2010). The S-O-R analytical framework has been widely applied to the research on the effect of environmental factors on customer value co-creation behavior and citizenship behavior (Zhu et al., 2016; Aljarah and Alrawashdeh, 2020). A social network is an environment for and carrier of TOCB. Social support from social networks is advantageous to tourists in optimizing tourism decisions and consumption experience and improving their sense of self-determination, leading to behavioral reactions. Thus, social support can be regarded as a stimulus, a sense of self-determination can be regarded as an organism, and TOCB can be regarded as a response. This study therefore discusses the effect of social support on TOCB through the sense of self-determination by constructing an S-O-R model, as shown in Figure 1.

### Research assumption

#### Social support and sense of self-determination

According to self-determination theory, if the external environment benefits from achieving expected goals, individuals’ sense of self-determination would be strengthened; if not, it would be weakened (Deci and Ryan, 2000). On their trip, tourists obtain travel information and emotional support from relatives, friends, and the organizations or individuals they contact through social media. The social network has become a kind of external environment that helps to optimize tourism experiences for tourists and has an important connection with tourists’ sense of self-determination. As a kind of valuable information, social support makes individuals feel cared for, loved, and respected and realize that they are members of a social network (Cobb, 1976).

Interactional communication is the primary way for individuals to get social support during consumption (Kang and Ridgway, 1996). On the one hand, social interaction can cultivate individuals’ sense of self-concern, self-adjustment, and connection with others; it can also meet individuals’ basic psychological needs for autonomy, competence, and relatedness (Ryan and Solky, 1996). On the other hand, the content of people’s communication is primarily related to consumer products and related knowledge, which can promote the acquisition of individual knowledge and enhance their perceived competence (Hsu et al., 2007; Kim et al., 2011). In other words, information support enables recipients to acquire knowledge and abilities, which is favorable for them to complete specific tasks and promote their perceived competence (Köhler et al., 2011). Because of the characteristics of tourism activities, such as advance purchase and consumption in different places, the decision-making of tourism consumption is fraught with uncertainty. To reduce decision risks, people tend to rely on the independent and fair tourism information the experienced provide, especially the comments, opinions, and advice from relatives and friends and other members of social networks, which are viewed as objective and reliable information sources (Kang and Schuett, 2013). Therefore, as soon as tourists get information support when making tourism decisions, they will develop a sense of competence and control over tourism decisions and consumption behavior. Following these arguments, the following hypotheses are advanced:

*Hypothesis 1a:* Information support has a positive impact on tourist perceived autonomy.

*Hypothesis 1b:* Information support has a positive impact on tourist perceived competence.

*Hypothesis 1c:* Information support has a positive impact on tourist perceived relatedness.

Social support can bring warmth to people, promote mutual understanding, and satisfy psychological needs (Liang et al., 2011). Both information and emotional support can enable individuals to develop perceived self-value, satisfaction, and relatedness. If individuals can take advantage of this social support, they will increase their sense of control over their life (Song and Fan, 2013). In essence, emotional support refers to spiritual support, care, and encouragement that help to enhance the understanding of intimacy between individuals and others and strengthen their sense of membership and belonging. Even if the support and maintenance of others felt by individuals in the social interaction cannot provide practical help for directly solving problems, they can make the individuals feel cared for (Chen et al., 2013). At the same time, as emotional support can make individuals think of spiritual support from others, it also can help indirectly enhance the ability of individuals to solve problems to improve their perceived competence (Liang et al., 2011). This spiritual support and encouragement can make individuals feel their tasks are from their heart instead of being imposed, thus helping to enhance their perceived autonomy. In the context of tourism consumption, particularly emotional support such as followers, likes, and comments from tourist social networks can make tourists develop emotional resonance and feel proud after making informed tourism decisions, stimulate tourists’ confidence for gaining expected tourism experience and strengthen tourists’ recognition of social network membership. Based on this, the study puts forward the following research hypotheses:

*Hypothesis 2a:* Emotional support has a positive impact on tourist perceived autonomy.

*Hypothesis 2b:* Emotional support has a positive impact on tourist perceived competence.

*Hypothesis 2c:* Emotional support has a positive impact on tourist perceived relatedness.

#### Sense of self-determination and TOCB

TOCB is a kind of spontaneous extra-role behavior, and emotionally positive individuals tend to have extra-role behavior (Hsieh and Chang, 2016). In tourism activities, tourists maintain constant social interaction and association and are in a state of social exchange. If tourists benefit from others, achieving autonomy, competence, and relatedness, they will generate positive attitudes and behavior that reward others, such as information sharing, service recommendation, and helping others, all of which constitute TOCB. Greguras and Diefendorff (2009) think that the sense of self-determination can influence individuals’ willingness and sense of identification which are embedded in organizational relationships, and promote individuals’ positive value co-creation in the interaction. Perceived autonomy and competence positively affect the positive experience, which accelerates the formation of extra-role behavior (Greguras and Diefendorff, 2009). From this, tourists with a higher sense of self-determination more easily reach an internally driven state and show a higher level of participation, interest, and behavior in social exchange. In addition, according to other fields of empirical results, the sense of self-determination positively affects customers’ citizenship behavior through improving personal connections (Chiu et al., 2019). In the brand community, customers’ perceived competence and connection help enhance community promises and customer citizenship behavior (Fu et al., 2018). According to these arguments, the following hypotheses are suggested:

*Hypothesis 3a:* Perceived autonomy has a positive impact on TOCB.

*Hypothesis 3b:* Perceived competence has a positive impact on TOCB.

*Hypothesis 3c:* Perceived relatedness has a positive impact on TOCB.

#### The intermediary role of sense of self-determination.

The S-O-R analysis framework provides a reasonable theoretical explanation for individuals’ reaction behavior formed under environmental factors. According to the framework, individuals do not respond passively to the stimulus in the external environment but make choices and reply through their own selective internal processing. When individuals perceive support from others, they will feel responsible for supporting others, and the direction and intensity of such behaviors are closely related to their benefits (Crocker and Canevello, 2008). With the richness of materials, the strengthening of the value of power, and the enhancement of subject consciousness, an increasing number of tourists are not willing to accept homogeneous tourism products passively but strive to dominate tourism activities and pursue personalized and high-quality tourism experiences. This goal cannot be achieved without information and emotional support from the social network. During the tour, the more social support tourists receive, the stronger their sense of self-determination and control over the content of tourism activities and the service delivery process. Out of the internal need to balance social exchange relations, tourists receiving a stronger sense of self-determination are more willing to devote personal resources such as time, energy, and knowledge to providing feedback, support, and help. It can be seen that the sense of self-determination plays a mediating role in the effect of social support on TOCB. According to these arguments, the following hypotheses are proposed:

*Hypothesis 4a:* Perceived autonomy mediates the relationship between information support and TOCB.

*Hypothesis 4b:* Perceived competence mediates the relationship between information support and TOCB.

*Hypothesis 4c:* Perceived relatedness mediates the relationship between information support and TOCB.

*Hypothesis 5a:* Perceived autonomy mediates the relationship between emotional support and TOCB.

*Hypothesis 5b:* Perceived competence mediates the relationship between emotional support and TOCB.

*Hypothesis 5c:* Perceived relatedness mediates the relationship between emotional support and TOCB.

## Materials and methods

### Measures

The questionnaire consists of two parts. The first part measures latent variables such as information support (IS), emotional support (ES), perceived autonomy (PA), perceived competence (PC), perceived relatedness (PR), and TOCB. The second part describes the respondents’ gender, age, occupation, education level, and monthly income. All variables in this study were derived from well-established scales provided in Appendix 1. The items of IS (3 items) and ES (4 items) were adopted from Liang et al. (2011). The items of PA (3 items), PC (3 items), and PR (3 items) were derived from Johnston and Finney (2010). TOCB is conceived as a reflective second-order construct formed of sharing (SH), help (HE), and recommendation (RE). SH was measured by three items derived from Oliveira et al. (2020). HE and RE were measured by three items from Li et al. (2022). To ensure measurement validity, all items were translated into Chinese using a blind translation–back–translation method. Respondents were asked to assess their level of agreement on each item using a 7-point Likert scale (1 = *strongly disagree*; 7 = *strongly agree*).

### Data collection and sampling

The respondents are aged over 15 and people who had traveled within the last 6 months were surveyed. All questionnaires were distributed online due to the COVID-19 epidemic, and the distribution time spanned from 12 to 26 October 2021. The snowballing method was introduced to collect data from individual social networks. Questionnaires were published on the Questionnaire Star survey platform. The respondents were given the website address and made a progress on the questionnaire by clicking or touching the answers on the screen. Some scholars believe that a questionnaire survey conducted through personal social relations in the Chinese context can better obtain the cooperation of respondents, thus ensuring the filling quality and recovery rate of questionnaires (Hitt et al., 2004). Moreover, this snowballing questionnaire survey has been used many times and proved to be a better data collection method (Hennig-Thurau et al., 2002; Chan and Kuok, 2021). Especially in the COVID-19 pandemic, this kind of non-personnel direct contact survey is more maneuverable.

The survey was self-administered and distributed to 412 respondents. Among these, 35 responses were excluded due to missing data or duplicate cases. The remaining sample of 377 responses was used for the analyses. Among the respondents, 50.7% were female and 49.3% were male. The most significant percentage (46.6%) was 21–40 years old and 41.9% were university/college graduates. In all, 38.7% of the respondents were employees of companies and 17.5% had a job in government or public institution. Respondents earning a monthly income of US $1155–1,443 represented 33.7%, and the group making a monthly income of US $866–1,154 accounted for 28.1%. Respondents’ profiles are summarized in Table 1.

**Table 1:** The demographic characteristics (*N* = 377).

Variable	Category	Frequency (*N*)	%
Gender	Male	186	49.3
	Female	191	50.7
Age	15–20	11	2.9
	21–40	175	46.4
	41–60	158	41.9
	>60	33	8.8
Education	Haven’t completed high school	12	3.2
	High school/secondary school	49	13.0
	Junior college	85	22.5
	University/college	158	41.9
	Master’s degree or above	73	19.4
Profession	Student	22	5.8
	Government employee/public institution staff	66	17.5
	Employee of company	146	38.7
	Laborer	18	4.8
	Individual business	65	17.2
	Freelancer	44	11.7
	Other	16	4.2
Income level (1 RMB = 0.1443 USD)	Below US $577	13	3.4
	US $577–865	32	8.5
	US $866–1,154	106	28.1
	US $1155–1,443	127	33.7
	US $1,444 or above	99	26.3

## Results

### Common method variance

To reduce the impact of common method variance (CMV), we conducted preventive control by optimizing the order of items and asking respondents to complete the questionnaire anonymously. Nonetheless, the potential CMV may have contaminated the correlations or path coefficients due to all self-reported data under the cross-sectional research design. For this reason, Harman’s single-factor test was utilized to assess the presence of CMV (Podsakoff et al., 2003). The results of factor analysis indicated that the one-factor model explained 36.02% of the variance, while the results of the eight-factor model indicated that 75.15% of the variance was explained by the eight underlying factors. Thus, CMV did not cause severe problems in this study.

### Reliability and validity evaluation

To determine the reliability of the measurement scales, Cronbach’s α and composite reliability (CR) values were computed for each construct. All Cronbach’s α and CR values surpassed the cut-off of 0.70. The measures in this study were accordingly reliable.

The modified scale to measure TOCB in this study was examined in a second order as Torres-Moraga et al. (2021) initially did. Before estimating the whole measurement model, a separate second-order confirmatory factor analysis (CFA) for the TOCB scale was conducted based on the suggestions of Bollen and Stine (1992). We performed this analysis using the SPSS Amos 24 package and employed a maximum likelihood approach. The results show that the fit indices are acceptable (χ^2^/df = 1.587, GFI = 0.978, AGFI = 0.959, CFI = 0.990, TLI = 0.985, RMSEA = 0.040). As depicted in Table 2, the path coefficients of the three first-order factors (sharing, help, and recommendation) and the second-order factor (TOCB) are higher than 0.70, and the variance explanation rates are all over 50%. In addition, bootstrap analysis results (extraction number is 5,000, 95% confidence interval), the standard error and deviation estimates of the first-order factor load are minimal (<0.001), which validated that TOCB was a second-order construct (Dwivedi and Merrilees, 2016).

**Table 2:** Results of second order model.

Construct	Path coefficient	*t-*statistic	Bias-corrected confidence intervals (BCCIs)	
			5%	95%
TOCB → SH	0.770	10.53	0.645	0.883
TOCB → HE	0.774	11.20	0.637	0.885
TOCB → RE	0.724	10.28	0.622	0.809

Then, following Bollen and Stine (1992), the whole measurement model was performed, and the goodness-of-fit indices (χ^2^/df = 1.337, GFI = 0.936, AGFI = 0.916, CFI = 0.983, TLI = 0.979, RMSEA = 0.030) supported an acceptable fit (Bentler, 1990). The standardized factor loading values of each measurement item range from 0.718 to 0.872 and are significant at the level of 0.001 (see Table 3), indicating that the measurement scale has good aggregation validity. Additionally, all constructs’ average variance extracted (AVE) ranged from 0.570 to 0.699, higher than 0.50, also supporting the satisfaction of the convergent validity of scales (Fornell and Larcker, 1981). Further, the correlation coefficients between each variable and others are calculated, and their absolute values are compared with the square roots of AVEs to judge the discriminant validity of the scale. As presented in Table 4, the correlation coefficient between variables ranges from 0.310 to 0.600, and the square root of the AVEs value range from 0.755 to 0.836. The square roots of AVEs were higher than the correlation coefficients between variables, thus implying satisfactory discriminant validity (Fornell and Larcker, 1981).

**Table 3:** Results of measurement model.

Construct		Item	Std. loading	*t*-statistic	CR	AVE	α
Information support		IS1	0.753	15.58	0.799	0.570	0.798
		IS2	0.792	16.64			
		IS3	0.718	14.68			
Emotional support		ES1	0.843	19.50	0.884	0.657	0.882
		ES2	0.868	20.43			
		ES3	0.728	15.77			
		ES4	0.797	17.95			
Perceived autonomy		PA1	0.800	17.18	0.828	0.616	0.827
		PA2	0.756	15.94			
		PA3	0.797	17.08			
Perceived competence		PC1	0.786	17.39	0.874	0.699	0.873
		PC2	0.848	19.35			
		PC3	0.872	20.14			
Perceived relatedness		PR1	0.784	17.00	0.838	0.633	0.838
		PR2	0.797	17.41			
		PR3	0.806	17.67			
TOCB	Sharing	SH1	0.747	15.59	0.804	0.579	0.801
		SH2	0.810	17.34			
		SH3	0.722	14.93			
	Help	HE1	0.794	16.98	0.817	0.598	0.814
		HE2	0.785	16.74			
		HE3	0.740	15.50			
	Recommendation	RE1	0.762	16.34	0.835	0.628	0.834
		RE2	0.808	17.72			
		RE3	0.807	17.67			

**Table 4:** Descriptive statistics and correlation estimates.

Variable	Mean	SD	IS	ES	PA	PC	PR	SH	HE	RE
IS	5.64	0.647	0.755							
ES	5.66	0.820	0.390^**^	0.811						
PA	5.04	0.842	0.417^**^	0.447^**^	0.785					
PC	5.36	0.845	0.489^**^	0.310^**^	0.414^**^	0.836				
PR	5.58	0.757	0.524^**^	0.578^**^	0.558^**^	0.394^**^	0.796			
SH	5.82	0.738	0.576^**^	0.365^**^	0.534^**^	0.437^**^	0.529^**^	0.761		
HE	5.60	0.605	0.555^**^	0.434^**^	0.478^**^	0.457^**^	0.536^**^	0.600^**^	0.773	
RE	5.05	0.811	0.497^**^	0.588^**^	0.456^**^	0.453^**^	0.573^**^	0.554^**^	0.562^**^	0.792

Diagonal values represent square root values of the AVE.

** *p* < 0.01.

### Structural model and hypotheses testing

#### Path analysis

Structural equation modeling (SEM) and maximum likelihood estimation were conducted to test the causal interconnectedness between research variables in AMOS 24. Overall, the goodness-of-fit indices (χ^2^/df = 1.612, GFI = 0.917, AGFI = 0.900, CFI = 0.966, TLI = 0.962, RMSEA = 0.040) support an acceptable fit of the structural model (Schumacker and Lomax, 2004). As presented in Table 5, all hypotheses are empirically supported. Specifically, information support has positive effects on perceived autonomy (H1a supported: β = 0.347, *p* < 0.01), perceived competence (H1b supported: β = 0.466, *p* < 0.01), and perceived relatedness (H1c supported: β = 0.403, *p* < 0.01). Similarly, emotional support is positively related to perceived autonomy (H2a supported: β = 0.337, *p* < 0.01), perceived competence (H2b supported: β = 0.145, *p* < 0.05), and perceived relatedness (H2c supported: β = 0.448, *p* < 0.01). Furthermore, perceived autonomy (H3a supported: β = 0.307, *p* < 0.01), perceived competence (H3b supported: β = 0.314, *p* < 0.01), and perceived relatedness (H3c supported: β = 0.491, *p* < 0.01) are found to be significant drivers of TOCB.

**Table 5:** Results of path analysis.

Hypothesis	Relationships	Path coefficient	*t*-statistic	Supported?
H1a	IS → PA	0.347^**^	5.34	Yes
H1b	IS → PC	0.466^**^	6.87	Yes
H1c	IS → PR	0.403^**^	6.59	Yes
H2a	ES → PA	0.337^**^	5.53	Yes
H2b	ES → PC	0.145^*^	2.48	Yes
H2c	ES → PR	0.448^**^	7.74	Yes
H3a	PA → TOCB	0.307^**^	4.99	Yes
H3b	PC → TOCB	0.314^**^	5.36	Yes
H3c	PR → TOCB	0.491^**^	6.93	Yes

* *p* < 0.05;

** *p* < 0.01.

#### Mediation effect testing emotional support

This study used bootstrapping to examine the mediating effects of perceived autonomy, competence, and relatedness with the help of Mplus 7.4. With reference to Li et al. (2022), the bootstrapping sample was set as 2,000, and the bias-corrected confident interval was set as 95%. Table 5 outlines the results of testing for mediation. The indirect influences of information support on TOCB through the proposed three paths are all significantly supported, given that the 95% bias-corrected confident interval excludes 0 (Lau and Cheung, 2012). The results imply that perceived autonomy, perceived competence, and perceived relatedness play mediating roles in the relationship between information support and TOCB. Thus, H4a to H4c are empirically supported. Table 6 depicts that the path coefficients of emotional support indirectly affecting TOCB are 0.046 to 0.220, respectively, and the 95% confidence interval also excludes 0, indicating that perceived autonomy, perceived competence, and perceived relatedness significantly mediate the relationship between emotional support and TOCB. Thus, H5a to H5c are empirically supported.

**Table 6:** Results of mediating effects.

Hypothesis	Relationships	Indirect effects	SE	*t*-statistic	BCCIs		Supported?
					5%	95%	
H4a	IS → PA → TOCB	0.107	0.036	3.00	0.055	0.173	Yes
H4b	IS → PC → TOCB	0.146	0.042	3.53	0.087	0.224	Yes
H4c	IS → PR → TOCB	0.198	0.051	3.91	0.124	0.290	Yes
H5a	ES → PA → TOCB	0.104	0.034	3.05	0.056	0.169	Yes
H5b	ES → PC → TOCB	0.046	0.025	1.85	0.013	0.094	Yes
H5c	ES → PR → TOCB	0.220	0.043	5.13	0.158	0.299	Yes

## Conclusion and implications

### Conclusion

Based on the self-determination theory and S-O-R analysis framework, this study constructed a theoretical model of the effect of social support on TOCB through the sense of self-determination, which was empirically tested with 377 sample data. It is found that social support has a significant positive effect on TOCB through the sense of self-determination, which plays a mediating role in the relationship between social support and TOCB.

Although the driving factors of TOCB have not specifically been addressed in previous literature, our results are consistent with those of other studies that have examined related concepts. For example, our results echo those of Rosenbaum and Massiah’s (2007), who found that social support has a significant positive impact on customer citizenship behavior. In addition, the findings show that the relationship between social support and customer citizenship behavior is verified in tourism consumption. Furthermore, the result supports the research findings of Ryan and Solky (1996) and Xie et al. (2019). As a social resource that provides information and emotion, social support can satisfy individuals’ basic psychological needs for autonomy, competence, and connection and help to cultivate individuals’ self-esteem, self-regulation, and sense of connection (Ryan and Solky, 1996). In the era of mobile internet, every individual who comprehends the information and consumption experience plays an increasingly important role as the provider of social support. Social network members, in particular, can provide more objective and reliable information resources and support individuals to obtain psychological benefits. When making consumption decisions, tourists seek social support from social network members to improve their perceived competence and self-determination. According to social exchange theory, an individual’s behavior toward another individual does not arise out of the individual’s response based on self-interest.

Some studies have shown that perceived benefits positively affect tourist citizenship behavior in the virtual community (Xie et al., 2019). This reciprocal behavior is not only reflected in the social interaction between members of the virtual tourism community. When tourists obtain psychological benefits from social support, they will also think that they are responsible for providing positive feedback to the source of social support, thus generating tourist citizenship behavior that benefits related individuals. Therefore, the findings of this study are consistent with social exchange theory. TOCB can be regarded as a reward for tourists to improve their sense of self-determination through social support. Enhancing social support can improve tourists’ sense of self-determination and TOCB.

### Theoretical implications

This study makes theoretical contributions to previous literature in three ways. Firstly, it contributes to the literature on tourist citizenship behavior. Despite the fact that some scholars recognize the multidimensional nature of tourist citizenship behavior, scant research has conducted targeted research on one of these dimensions. Previous studies have paid attention to tourist citizenship behavior at the destination level (Torres-Moraga et al., 2021) and those in group package tours (Liu and Tsaur, 2014). Still, the antecedents and consequences of TOCB behavior remain underexplored. This study enhances our understanding of TOCB by finding that social support motivates TOCB by inducing the sense of self-determination.

Secondly, this study verifies that social support is an important antecedent of TOCB, which extends and expands the research on driving factors of TOCB. Social relations are invisible but constitute the environment and objective conditions for tourist citizenship behavior. However, early studies have not clearly understood whether and how social support significantly affects tourist citizenship behavior. Although relevant research has identified the driving factors of customer citizenship behavior, the connotation of customer citizenship behavior in tourism now presents new characteristics. This study responds to this problem by extending customer citizenship behavior to the tourism context, focusing on TOCB that is more closely related to social support received by tourists, and empirically testing the impact of social support on TOCB.

Thirdly, the most notable contribution of this study lies in its exploration of the mechanism of social support influencing TOCB. Tourist citizenship behavior is a spontaneous extra-role behavior, in essence, independent of the service delivery process and enterprise incentive system. It is essential to explore how tourist citizenship behavior comes into being from the point of view of individual psychology. Based on self-determination theory, relying on the S-O-R analysis framework, this study brings social support, a sense of self-determination, and TOCB into the same research framework and empirically tests the intermediary role of the sense of self-determination in the relationship between social support and TOCB, which not only opens the “black box” of the relationship among the three, but also provides a new perspective for understanding the mechanism that generates TOCB. In addition, linking the sense of self-determination and TOCB not only responds to the future research directions regarding the positive psychology applied in the tourism setting as suggested by Filep and Laing (2019), but also answers Zhu et al.’s (2016) call to empirically demonstrate the influence mechanism of psychological variables on customer citizenship behavior.

### Practical implications

The findings of this study can help tourism enterprises understand the influence of social support and the sense of self-determination on TOCB to provide a reference for the development of marketing strategy. This study makes the following contributions. First, tourism enterprises should understand and view the role of social support in enterprise and product information publicity. TOCB is not an isolated individual behavior but is deeply influenced by tourist social network, showing strong imitativeness and transmissibility. Therefore, tourism enterprises should engage in relationship marketing, establish and maintain a good relationship with tourists and the public, use the ripple effect of tourists on their associated individuals, and constantly improve the influence and competitiveness of tourism enterprises.

In addition, promoting tourists’ sense of self-determination is an important strategy to encourage TOCB. TOCB is a kind of autonomous behavior, and the sense of self-determination is an important driving factor of this behavior. Tourism enterprises should change their role, positioning from the leader of tourism activities to the participant in the process of co-creation of tourist experience value, support and help tourists to devote their efforts in the process of product design and service delivery according to their own will and way, and make them get a sense of self-determination from it, to promote their TOCB.

Finally, it is necessary to formulate appropriate incentive mechanisms to guide and stimulate TOCB. Studies have shown that extrinsic motivations such as obtaining additional rewards, maintaining relationships, and gaining praise are essential in sharing consumption experience (Lee et al., 2019). Tourism enterprises can set up incentive mechanisms such as star-rated membership, accessible experience, or rewards based on bonus points to encourage tourists to generate extra-role behavior such as sharing information, recommending services, helping others, and giving full play to the role of “part-time employees.”

## Limitations and future studies

Although this study arrives at several meaningful conclusions, the following limitations need to be addressed. One limitation is that the sample coverage was not comprehensive enough. This study used a personal social network to obtain sample data by the snowballing method. The respondents were mainly from some cities in China, and the sample was not representative enough, thus affecting the reliability of conclusions to a certain extent. In addition, in the survey process, the respondents were asked to recall their previous travel experience and complete the questionnaire based on it. Although this method is often used in many studies, the passage of time delay has caused a discrepancy between the respondents’ recall and their real feelings at the time. Finally, this study generally explored the effect of social support on TOCB without considering the possible moderating effects of tourists’ personality characteristics and types of tourism activities. Future research should improve the survey methods, enhance the sample coverage, and explore the driving factors and mechanisms of TOCB for different types of tourism activities and other forms of tourist participation.

## Data availability statement

The original contributions presented in the study are included in the article/supplementary material, further inquiries can be directed to the corresponding author.

## Author contributions

All authors listed have made a substantial, direct, and intellectual contribution to the work and approved it for publication.

## Funding

This work was supported by the Humanities and Social Sciences Youth Foundation, Ministry of Education of the People’s Republic of China (no. 19YJC630083).

## Conflict of interest

The authors declare that the research was conducted in the absence of any commercial or financial relationships that could be construed as a potential conflict of interest.

## Publisher’s note

All claims expressed in this article are solely those of the authors and do not necessarily represent those of their affiliated organizations, or those of the publisher, the editors and the reviewers. Any product that may be evaluated in this article, or claim that may be made by its manufacturer, is not guaranteed or endorsed by the publisher.

## Figures and Tables

**Table:** 

Items and statements	Sources
**Information support (IS)**	Liang et al. (2011)
IS1: Some people offered help when I needed it during this trip	
IS2: When I encountered a problem during this trip, some people gave me information to overcome it	
IS3: When faced with difficulties during this trip, some people gave me suggestions	
**Emotional support (ES)**	Liang et al. (2011)
ES1: When faced with difficulties during this trip, some people were on my side with me	
ES2: When faced with difficulties during this trip, some people comforted and encouraged me	
ES3: When faced with difficulties during this trip, some people listened to me talk about my private feelings	
ES4: When faced with difficulties during this trip, some people expressed concern for me	
**Perceived autonomy (PA)**	Johnston and Finney (2010)
PA1: I was free to decide for myself what to do during this trip	
PA2: I was free to express my ideas and opinions during this trip	
PA3: I felt like I was myself during this trip	
**Perceived competence (PC)**	Johnston and Finney (2010)
PC1: People I knew told me I was good at what I did during this trip	
PC2: I felt a sense of accomplishment from what I did during this trip	
PC3: I got much of a chance to show how capable I was during this trip	
**Perceived relatedness (PR)**	Johnston and Finney (2010)
PR1: I got along with people I came into contact with during this trip	
PR2: The people I interacted with regularly seemed to like me much during this trip	
PR3: People were generally pretty friendly toward me during this trip	
**Sharing (SH)**	Oliveira et al. (2020)
SH1: I shared photos or videos about this trip	
SH2: I shared personal blogs about this trip	
SH3: I shared reviews on WeChat or online platforms about this trip	
**Help (HE)**	Li et al. (2022)
HE1: I assisted others if they needed help with their trip	
HE2: I helped others if they seemed to have problems with their trip	
HE3: I gave others advice about their trip	
**Recommendation (RE)**	Li et al. (2022)
RE1: I said positive things about this trip to others	
RE2: I recommended something about this trip to others	
RE3: I encouraged friends and relatives to choose a similar trip	

## Appendix

**Appendix 1** Measurement scales.

## References

[ref1] Aicher T. J. Brenner J. Aicher T. J. (2015). Individuals’ motivation to participate in sport tourism: a self-determination theory perspective. Int. J. Sport Manage. Recreat. Tourism 18, 56–81. doi: 10.5199/ijsmart-1791-874X-18d

[ref2] Albrecht T. L. Adelman M. B. (1987). Communicating Social Support. Thousand Oaks, CA: Sage.

[ref3] Aljarah A. Alrawashdeh M. (2020). Boosting customer citizenship behavior through corporate social responsibility: does perceived service quality matter? Soc. Respons. J. 17, 631–647. doi: 10.1108/srj-03-2019-0098

[ref4] Arica R. Çorbaci A. (2020). The mediating role of the tourists’ citizenship behavior between the value co-creation and satisfaction. Adv. Hospital. Tourism Res. 8, 125–150. doi: 10.30519/ahtr.649639

[ref5] Assiouras I. Skourtis G. Giannopoulos A. Buhalis D. Koniordos M. (2019). Value co-creation and customer citizenship behavior. Ann. Tour. Res. 78:102742. doi: 10.1016/j.annals.2019.102742

[ref6] Bentler P. M. (1990). Comparative fit indexes in structural models. Psychol. Bull. 107, 238–246. doi: 10.1037/0033-2909.107.2.238, PMID: 2320703

[ref7] Bollen K. A. Stine R. A. (1992). Bootstrapping goodness-of-fit measures in structural equation models. Sociol. Methods Res. 21, 205–229. doi: 10.1177/0049124192021002004

[ref8] Bosnjak M. Brown C. A. Lee D. J. Yu G. B. Sirgy M. J. (2016). Self-expressiveness in sport tourism: determinants and consequences. J. Travel Res. 55, 125–134. doi: 10.1177/0047287514535845

[ref9] Chan S. H. J. Kuok O. M. K. (2021). Antecedents of civic virtue and altruistic organizational citizenship behavior in Macau. Soc. Bus. Rev. 16, 113–133. doi: 10.1108/SBR-06-2020-0085

[ref10] Chen A. Lu Y. Wang B. Zhao L. Li M. (2013). What drives content creation behavior on SNSs? A commitment perspective. J. Bus. Res. 66, 2529–2535. doi: 10.1016/j.jbusres.2013.05.045

[ref11] Chen S. C. Raab C. Tanford S. (2015). Antecedents of mandatory customer participation in service encounters: an empirical study. Int. J. Hosp. Manag. 46, 65–75. doi: 10.1016/j.ijhm.2015.01.012

[ref12] Cheng J. C. Wu C. S. Yen C. H. Chen C. Y. (2016). Tour leader attachment and customer citizenship behaviors in group package tour: the role of customer commitment. Asia Pac. J. Tourism Res. 21, 642–657. doi: 10.1080/10941665.2015.1068192

[ref13] Chiu C. M. Huang H. Y. Cheng H. L. Sun P. C. (2015). Understanding online community citizenship behaviors through social support and social identity. Int. J. Inf. Manag. 35, 504–519. doi: 10.1016/j.ijinfomgt.2015.04.009

[ref14] Chiu T. S. Ortiz J. Chih W. H. Pang L. C. Huang J. J. (2019). Antecedents of consumers’ citizenship behaviour towards organic foods. J. Consum. Behav. 18, 332–349. doi: 10.1002/cb.1774

[ref15] Chung N. Tyan I. Chung H. C. (2017). Social support and commitment within social networking site in tourism experience. Sustainability 9:2102. doi: 10.3390/su9112102

[ref16] Cobb S. (1976). Social support as a moderator of life stress. Psychosom. Med. 38, 300–314. doi: 10.1097/00006842-197609000-00003 981490

[ref17] Crocker J. Canevello A. (2008). Creating and undermining social support in communal relationships: the role of compassionate and self-image goals. J. Pers. Soc. Psychol. 95, 555–575. doi: 10.1037/0022-3514.95.3.555, PMID: 18729694

[ref18] Deci E. L. Ryan R. M. (2000). The “what” and “why” of goal pursuits: human needs and the self-determination of behavior. Psychol. Inq. 11, 227–268. doi: 10.1207/S15327965PLI1104_01

[ref19] Dwivedi A. Merrilees B. (2016). Holistic consumer evaluation of retail corporate brands and impact on consumer loyalty intentions. Australas. Mark. J. AMJ 24, 69–78. doi: 10.1016/j.ausmj.2016.02.002

[ref20] Engström J. Elg M. (2015). A self-determination theory perspective on customer participation in service development. J. Serv. Mark. 29, 511–521. doi: 10.1108/JSM-01-2015-0053

[ref21] Filep S. Laing J. (2019). Trends and directions in tourism and positive psychology. J. Travel Res. 58, 343–354. doi: 10.1177/0047287518759227

[ref22] Fornell C. Larcker D. F. (1981). Evaluating structural equation models with unobservable variables and measurement error. J. Market. Res. 18, 39–50. doi: 10.1177/002224378101800104

[ref23] Frías Jamilena D. M. Polo Pena A. I. Rodriguez Molina M. A. (2017). The effect of value-creation on consumer-based destination brand equity. J. Travel Res. 56, 1011–1031. doi: 10.1177/0047287516663650

[ref24] Fu J. Ko P. C. Lu C. Lee W. L. (2018). Brand engagement and co-creation in the online environments-based on the self-determination theory. In *Proceedings of the 2018 international conference on E-business and Mobile commerce* (pp. 11–16).

[ref25] Gagné M. Deci E. L. (2005). Self-determination theory and work motivation. J. Organ. Behav. 26, 331–362. doi: 10.1002/job.322

[ref26] Granovetter M. (1983). The strength of weak ties: a network theory revisited. Sociol Theory 1, 201–233. doi: 10.2307/202051

[ref27] Greguras G. J. Diefendorff J. M. (2009). Different fits satisfy different needs: linking person-environment fit to employee commitment and performance using self-determination theory. J. Appl. Psychol. 94, 465–477. doi: 10.1037/a0014068, PMID: 19271801

[ref28] Groth M. (2005). Customers as good soldiers: examining citizenship behaviors in internet service deliveries. J. Manag. 31, 7–27. doi: 10.1177/0149206304271375

[ref29] Hajli M. N. (2014). The role of social support on relationship quality and social commerce. Technol. Forecast. Soc. Chang. 87, 17–27. doi: 10.1016/j.techfore.2014.05.012

[ref30] Hennig-Thurau T. Gwinner K. P. Gremler D. D. (2002). Understanding relationship marketing outcomes: an integration of relational benefits and relationship quality. J. Serv. Res. 4, 230–247. doi: 10.1177/1094670502004003006

[ref31] Hitt M. A. Ahlstrom D. Dacin M. T. Levitas E. Svobodina L. (2004). The institutional effects on strategic alliance partner selection in transition economies: China vs. Russia. Organ. Sci. 15, 173–185. doi: 10.1287/orsc.1030.0045

[ref32] Hsieh S. H. Chang A. (2016). The psychological mechanism of brand co-creation engagement. J. Interact. Mark. 33, 13–26. doi: 10.1016/j.intmar.2015.10.001

[ref33] Hsu M. H. Ju T. L. Yen C. H. Chang C. M. (2007). Knowledge sharing behavior in virtual communities: the relationship between trust, self-efficacy, and outcome expectations. Int. J. Hum. Comput. Stud. 65, 153–169. doi: 10.1016/j.ijhcs.2006.09.003

[ref34] Huang J. Hsu C. H. (2010). The impact of customer-to-customer interaction on cruise experience and vacation satisfaction. J. Travel Res. 49, 79–92. doi: 10.1177/0047287509336466

[ref35] Hultman M. Skarmeas D. Oghazi P. Beheshti H. M. (2015). Achieving tourist loyalty through destination personality, satisfaction, and identification. J. Bus. Res. 68, 2227–2231. doi: 10.1016/j.jbusres.2015.06.002

[ref36] Johnston M. M. Finney S. J. (2010). Measuring basic needs satisfaction: evaluating previous research and conducting new psychometric evaluations of the basic needs satisfaction in general scale. Contemp. Educ. Psychol. 35, 280–296. doi: 10.1016/j.cedpsych.2010.04.003

[ref37] Kang Y. S. Ridgway N. M. (1996). The importance of consumer market interactions as a form of social support for elderly consumers. J. Public Policy Mark. 15, 108–117. doi: 10.1177/074391569601500110

[ref38] Kang M. Schuett M. A. (2013). Determinants of sharing travel experiences in social media. J. Travel Tour. Mark. 30, 93–107. doi: 10.1080/10548408.2013.751237

[ref39] Kim J. Song J. Jones D. R. (2011). The cognitive selection framework for knowledge acquisition strategies in virtual communities. Int. J. Inf. Manag. 31, 111–120. doi: 10.1016/j.ijinfomgt.2010.05.011

[ref40] Kim J. Tussyadiah I. P. (2013). Social networking and social support in tourism experience: the moderating role of online self-presentation strategies. J. Travel Tour. Mark. 30, 78–92. doi: 10.1080/10548408.2013.751220

[ref41] Klooster E. V. T. Go F. (2006). “Leveraging computer mediated communication for social support in educational travel” in Information and communication Technologies in Tourism (Vienna: Springer), p. 260.

[ref42] Köhler C. F. Rohm A. J. De Ruyter K. Wetzels M. (2011). Return on interactivity: the impact of online agents on newcomer adjustment. J. Mark. 75, 93–108. doi: 10.1509/jm.75.2.93

[ref44] Kumar J. Nayak J. K. (2019). Exploring destination psychological ownership among tourists: antecedents and outcomes. J. Hosp. Tour. Manag. 39, 30–39. doi: 10.1016/j.jhtm.2019.01.006

[ref45] Lau R. S. Cheung G. W. (2012). Estimating and comparing specific mediation effects in complex latent variable models. Organ. Res. Methods 15, 3–16. doi: 10.1177/1094428110391673

[ref46] Lee M. Lee J. Quilliam E. (2019). Motivations for sharing marketer-generated content on social media: a comparison between American and Korean college students. J. Consum. Mark. 36, 206–217. doi: 10.1108/JCM-07-2016-1875

[ref47] Li S. Chen G. Liu M. Xu J. Cao J. Yang J. (2022). How does tour guide humor influence tourist citizenship behavior? J. Hosp. Tour. Manag. 50, 108–118. doi: 10.1016/j.jhtm.2022.01.005

[ref48] Liang T. P. Ho Y. T. Li Y. W. Turban E. (2011). What drives social commerce: the role of social support and relationship quality. Int. J. Electron. Commer. 16, 69–90. doi: 10.2753/JEC1086-4415160204

[ref49] Liu L. Cui T. Wu J. Cao R. Ye Y. (2021). Encouraging tourist citizenship behavior through resource uniqueness and service quality: the mediating role of emotions. J. Vacat. Mark. 27, 45–60. doi: 10.1177/1356766720952101

[ref50] Liu J. S. Tsaur S. H. (2014). We are in the same boat: tourist citizenship behaviors. Tour. Manag. 42, 88–100. doi: 10.1016/j.tourman.2013.11.001

[ref51] Madjar N. (2008). Emotional and informational support from different sources and employee creativity. J. Occup. Organ. Psychol. 81, 83–100. doi: 10.1348/096317907X202464

[ref52] Namkung Y. Jang S. C. S. (2010). Effects of perceived service fairness on emotions, and behavioral intentions in restaurants. Eur. J. Mark. 44, 1233–1259. doi: 10.1108/03090561011062826

[ref53] Oliveira T. Araujo B. Tam C. (2020). Why do people share their travel experiences on social media? Tour. Manag. 78:104041. doi: 10.1016/j.tourman.2019.104041 PMC7168349 32322615

[ref54] Podsakoff P. M. MacKenzie S. B. Lee J. Y. Podsakoff N. P. (2003). Common method biases in behavioral research: a critical review of the literature and recommended remedies. J. Appl. Psychol. 88, 879–903. doi: 10.1037/0021-9010.88.5.879, PMID: 14516251

[ref55] Rather R. A. Najar A. H. Jaziri D. (2020). Destination branding in tourism: insights from social identification, attachment and experience theories. Anatolia 31, 229–243. doi: 10.1080/13032917.2020.1747223

[ref56] Rosenbaum M. S. Massiah C. A. (2007). When customers receive support from other customers: exploring the influence of intercustomer social support on customer voluntary performance. J. Serv. Res. 9, 257–270. doi: 10.1177/1094670506295851

[ref57] Ryan R. M. Deci E. L. (2017). Self-Determination Theory: Basic Psychological Needs in Motivation, Development, and Wellness. New York: Guilford Press.

[ref58] Ryan R. M. Solky J. A. (1996). “What is supportive about social support?” in Handbook of social support and the family (Boston, MA: Springer), 249–267.

[ref59] Schaefer C. Coyne J. C. Lazarus R. S. (1981). The health-related functions of social support. J. Behav. Med. 4, 381–406. doi: 10.1007/BF00846149 7338894

[ref1001] Schumacker R. E. Lomax R. G. (2004). A Beginner’s Guide to Structural Equation Modeling (Second Edition). Mahwah, NJ: Lawrence Erlbaum Associates Inc).

[ref60] Shafiee M. M. Tabaeeian R. A. Khoshfetrat A. (2020). Tourist engagement and citizenship behavior: the mediating role of relationship quality in the hotel industry. Tour. Hosp. Res. 20, 481–492. doi: 10.1177/1467358420914373

[ref61] Song J. Fan H. (2013). A meta-analysis of the relationship between social support and subjective well-being. Adv. Psychol. Sci. 21, 1357–1370. doi: 10.3724/SP.J.1042.2013.01357

[ref62] Thoits P. A. (1995). Stress, coping, and social support processes: where are we? What next? J. Health Soc. Behav. 35, 53–79. doi: 10.2307/2626957 7560850

[ref63] Torres-Moraga E. Rodriguez-Sanchez C. Sancho-Esper F. (2021). Understanding tourist citizenship behavior at the destination level. J. Hosp. Tour. Manag. 49, 592–600. doi: 10.1016/j.jhtm.2021.11.009

[ref64] Tsaur S. H. Yang T. L. Tsai C. H. (2021). Tour leader likeability and tourist citizenship behaviours: mediating effect of perceived value. Curr. Issue Tour. 24, 2628–2642. doi: 10.1080/13683500.2020.1849044

[ref65] Van der Poel M. G. (1993). Delineating personal support networks. Soc. Netw. 15, 49–70. doi: 10.1016/0378-8733(93)90021-C

[ref66] Verleye K. Gemmel P. Rangarajan D. (2014). Managing engagement behaviors in a network of customers and stakeholders: evidence from the nursing home sector. J. Serv. Res. 17, 68–84. doi: 10.1177/1094670513494015

[ref67] Wong I. A. Lin Z. (2022). Understanding tourist citizenship behavioral intentions: the role of social interactions and brand perceptions. J. China Tour. Res. 18, 592–610. doi: 10.1080/19388160.2021.1939829

[ref68] Xie L. Zhao Q. Ma K. (2019). Relationship among interaction, perceived benefits and citizenship behavior of virtual travel community members: from a value co-creation perspective. Tour. Tribune 34, 28–40.

[ref69] Yi Y. Gong T. Lee H. (2013). The impact of other customers on customer citizenship behavior. Psychol. Mark. 30, 341–356. doi: 10.1002/mar.20610

[ref70] Zhang H. Cheng Z. Chen X. (2022). How destination social responsibility affects tourist citizenship behavior at cultural heritage sites? Mediating roles of destination reputation and destination identification. Sustainability 14:6772. doi: 10.3390/su14116772

[ref71] Zhu D. H. Sun H. Chang Y. P. (2016). Effect of social support on customer satisfaction and citizenship behavior in online brand communities: the moderating role of support source. J. Retail. Consum. Serv. 31, 287–293. doi: 10.1016/j.jretconser.2016.04.013

